# Androgens and Their Role in Regulating Sex Differences in the Hypothalamic/Pituitary/Adrenal Axis Stress Response and Stress-Related Behaviors

**DOI:** 10.1089/andro.2021.0021

**Published:** 2021-12-23

**Authors:** Julietta A. Sheng, Sarah M.L. Tan, Taben M. Hale, Robert J. Handa

**Affiliations:** ^1^ Department of Biomedical Sciences, Colorado State University, Fort Collins, Colorado, USA.; ^2^ Department of Basic Medical Science, University of Arizona College of Medicine - Phoenix, Arizona, USA.

**Keywords:** androgen, estrogen, glucocorticoids, HPA axis, SARMs, androgen therapy

## Abstract

Androgens play a pivotal role during development. These gonadal hormones and their receptors exert organizational actions that shape brain morphology in regions controlling the stress regulatory systems in a male-specific manner. Specifically, androgens drive sex differences in the hypothalamic/pituitary/adrenal (HPA) axis and corresponding hypothalamic neuropeptides. While studies have examined the role of estradiol and its receptors in sex differences in the HPA axis and associated behaviors, the role of androgens remains far less studied. Androgens are generally thought to modulate the HPA axis through the activation of androgen receptors (ARs). They can also impact the HPA axis through reduction to estrogenic metabolites that can bind estrogen receptors in the brain and periphery. Such regulation of the HPA axis stress response by androgens can often result in sex-biased risk factors for stress-related disorders, such as anxiety and depression. This review focuses on the biosynthesis pathways and molecular actions of androgens and their nuclear receptors. The impact of androgens on hypothalamic neuropeptide systems (corticotropin-releasing hormone, arginine vasopressin, oxytocin, dopamine, and serotonin) that control the stress response and stress-related disorders is discussed. Finally, this review discusses potential therapeutics involving androgens (androgen replacement therapies, selective AR modulator therapies) and ongoing clinical trials.

## Introduction

Androgens exert many neurobiological effects, one of which is to regulate the hypothalamic function.^1,2^ In part, these actions occur through androgenic regulation of the hypothalamic/pituitary/adrenal (HPA) and the hypothalamic/pituitary gonadal axes, thereby influencing important neurobiological functions such as autonomic and neuroendocrine function, feeding and metabolism, and stress-related and reproductive behaviors.^3^ Acute exposure to glucocorticoids (GCs), the product of the activation of the HPA axis, can be beneficial and enhance cognition and increase metabolism, whereas chronic exposure can lead to cardiovascular disease, metabolic and feeding disorders, neurological disorders, and behavioral disruption.^4,5^

Both neuroendocrine axes are intertwined with changes in the levels of circulating GCs, in part, regulated by gonadal steroid hormone action on hypothalamic function. Gonadal hormones are further implicated as the underlying cause for sex differences in neurological disorders.^6^ Whereas many studies have examined the role of estrogens and estrogen receptors (ERs) in regulating the HPA axis and related behaviors,^7^ the role for androgens and androgen receptors (ARs) is not as widely explored.

The focus of this review is to examine the HPA axis function and related regulatory neuropeptide (arginine vasopressin [AVP], oxytocin [OT], corticotropin-releasing factor [CRH], serotonin [5-HT], and dopamine [DA]) expression and action as they relate to androgens.^7^ The role of androgens and ARs in stress-related disorders and potential therapeutic methods are discussed.^8^

## Molecular Actions of Androgens in the Brain

The sexual differentiation of the male phenotype is heavily driven by androgens. Androgens exert organizational actions during development to program lasting sex differences in the brain. There is a prenatal surge of testosterone (T) during late gestation (gestation day 18 in the rat) and a second surge that occurs immediately following parturition, both of which masculinizes and defeminizes the brain in males.^9–11^ Similarly, humans are exposed to a surge of T during gestation that also allows for sexual differentiation of the brain and development of sex differences in behavior and hormone release during development.^12^ T is produced primarily in the testis.

**FIG. 1.**
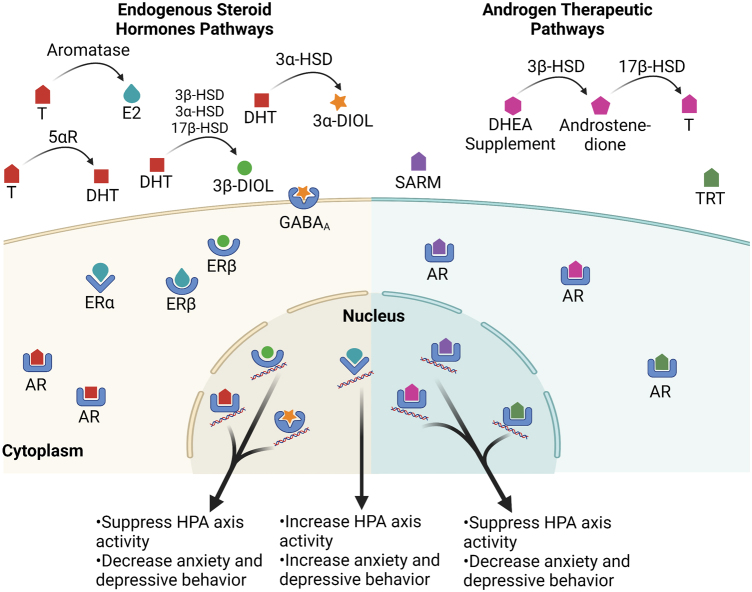
Endogenous steroid hormone pathways and androgen replacement therapies. T is converted to DHT and E2 by 5αR and aromatase, respectively. DHT is further metabolized to 3β-diol by 3β-HSD, 3α-HSD, or 17β-HSD, and 3α-diol by 3α-HSD. Both T and DHT bind AR in the cytoplasm and enter the nucleus to bind the DNA. E2 binds either ERα or ERβ. 3β-diol also binds ERβ while 3α-diol binds GABA_A_. ERα, ERβ, and GABA_A_ are translocated to the nucleus to bind DNA and drive changes to the HPA axis and related behavior. SARMs, TRT, and T derived from DHEA additionally bind cytoplasmic AR. These therapies also bind nuclear DNA to suppress HPA axis activity and decrease anxiety and depressive behaviors. Figure created with BioRender.com. 3α-diol, 3α-androstanediol glucuronide; 3β-diol, 5α-androstane-3β, 17β diol; 3α-HSD, 3α-hydroxysteroid; 3β-HSD, 3β-hydroxysteroid dehydrogenase; 5αR, 5α-reductase; 17β-HSD, 17β-hydroxysteroid dehydrogenase; ERα, estrogen receptor α; ERβ, estrogen receptor β; AR, androgen receptor; DHEA, dehydroepiandrosterone; DHT, dihydrotestosterone; E2, estradiol; GABA, gamma-aminobutyric acid; HPA, hypothalamic/pituitary/adrenal; SARM, selective androgen receptor modulator; T, testosterone; TRT, testosterone replacement therapy.

Dehydroepiandrosterone (DHEA) is first converted to 4-androstenedione by the enzyme 3β-hydroxysteroid dehydrogenase (3β-HSD), followed by the conversion to T by enzyme 17β-hydroxysteroid dehydrogenase (17β-HSD). T is then converted into estradiol (E2) by aromatase or into dihydrotestosterone (DHT) by 5α-reductase (5αR) in tissues in which these enzymes are expressed (Fig. 1). Dysregulations of such enzymes are implicated in various androgen-related disorders. Individuals with decreased expressions of 17β-HSD or 5αR lead to male pseudohermaphroditism present with ambiguous female virilization and external genitalia around puberty.^6^

The actions of androgens are largely mediated by the AR. AR is expressed in a wide range of tissues, with levels that vary during development and throughout the life span.^13^ AR is a member of a family of steroid nuclear receptors that share similar structural and functional identity.^14,15^ Nuclear receptors share a crystal structure composed of a β-sheet (S1/S2) and 12 α-helices (H1–H12). H4–H6 and H9 are found between H1–H3 on one end and H7 and H10–H11 on the other end.^16^ The first step in AR activation is the binding of a ligand (e.g., T) in the binding pocket of cytoplasmic AR. This activates AR by inducing release of several heat shock proteins. AR can then interact with the DNA at its specific androgen response element (ARE).

Interestingly, the consensus sequence of the ARE, GG(A/T)ACAnnnTGTTCT, is very similar to the consensus sequence of GC response elements (GREs). Therefore, when ARE is activated, the nearby GRE is also detected, implicating the interaction of GC receptor (GR), AR, and mineralocorticoid receptor (MR) with similar sequences.^17^ Once AR is bound to ARE on the DNA, components important for transcription are recruited. The recruitment of these components is mediated by the interaction between AR N-terminus, TATA box-binding protein, and TFIIF compound.^18^ Mechanisms of inhibition of AR actions are less understood. When AR is bound to an antagonist ligand, inhibitory proteins are recruited.

Such inhibitory proteins compete with coactivators of transcription, prevent the entry of AR into the nucleus to interact with the DNA, or induce binding of AR to the DNA.^19^ Specifically, coinhibitor, short heterodimer partner (SHIP), prevents AR from entering the nucleus by tethering it to the cytoplasm.^20^ Gobinet et al.^21^ hypothesize that SHIP also competes with coactivators and attracts additional inhibitory proteins to AR to inhibit it. Further research on inhibitory mechanisms of AR could allow better understanding of dysregulations between androgens and their receptor-mediated effects.

## Androgens Regulate the HPA Axis Stress Response in a Sex-Dependent Manner

### Sex-dependent regulation of HPA axis activation

The HPA axis is an intricate stressor-responsive system that allows central communication between hypothalamic neurons, the pituitary gland, and the adrenal glands in the periphery. Activation of the HPA axis occurs by afferent inputs to the paraventricular nucleus of the hypothalamus (PVN). While some inputs to the PVN arise from upstream extrahypothalamic and limbic regions through direct serotonergic or catecholaminergic pathways, others activate the PVN directly.^9^

Upon activation of the HPA axis, parvocellular neurons in the PVN synthesize several neuropeptides (CRH, AVP, and OT) that are released into the hypophyseal portal vasculature to drive secretion of adrenocorticotropic hormone (ACTH) from the anterior pituitary. Release of ACTH into the general circulation drives further secretion of GC synthesis and release from the adrenal cortex^22–25^ (cortisol in humans, and corticosterone [CORT] in rats and mice). Circulating GCs act upon peripheral tissue to elicit a myriad of effects. Acute stress-induced GC exposure is beneficial in that it improves cognition and augments physiological responses and energy required in the fight-or-flight response while simultaneously suppressing digestive and reproductive functions.

While short-term elevations of GCs are beneficial for physiological function and survival, chronic exposure to elevated GCs has the opposite effect. Long-term exposure to GCs suppresses immune and neural functions through inhibiting neuronal and glial resilience, glucose uptake, and energy balance, leading to neurotoxicity.^22,23^ Moreover, chronic elevations of GC can also alter social, anxiety-, depressive-like, and reproductive behaviors,^24,26^ increasing the risk for metabolic and neuropsychiatric disorders.^25,27^

Sex differences in HPA axis activity have been reported in numerous publications over the past 50 years. In rodents, females show a more robust and prolonged CORT and ACTH response to acute stressors than males.^28,29^ Levels of *Crh* mRNA in the PVN and the ACTH precursor protein, proopiomelanocortin, in the anterior pituitary are also greater in females. Together, such data indicate an enhanced HPA axis stress response and decreased GC negative feedback.^30^ At rest, females display higher baseline levels of CORT than males, suggesting increased basal HPA axis function.

Importantly, gonadectomy (GDX) of male and female rodents has been shown to decrease basal levels of CORT in females and raise levels in males. GDX with hormone replacement (E2 in females, T in males) reinstates the sex difference in CORT to that of intact animals, indicating a significant role of gonadal hormones in CORT secretion.^7^

Corticosteroid binding globulin (CBG) is another important player to consider when examining sex differences in HPA axis function. CBG is a circulating glycoprotein of liver origin, which binds circulating corticosteroids following their release by the adrenal gland. It is thought that the primary role of CBG is to prevent degradation of corticosteroid during transport to target tissues. At the target tissues, corticosteroids are released from CBG and can bind their intracellular receptors.^31,32^

Hence, CBGs directly regulate the availability of plasma free-CORT and its ability to act upon target tissues. Baseline CBG levels in females are shown to be twofold higher than in males, whereas bioavailable free CORT is not different between the two sexes.^33^ Therefore, increased levels of CBG could be acting as a buffer against the increased basal and stress-induced CORT found in females. It is also likely that the higher CBG levels in females may partly contribute to the blunted HPA axis negative feedback mechanism seen after an acute stressor since corticosteroids can only bind target tissues when not bound to CBG.^31,34^

### Sex-dependent regulation of negative feedback inhibition of the HPA axis

GC feedback inhibition on the HPA axis returns levels of adrenal hormones to baseline following stress-induced increases and prevents baseline CORT levels from getting too high or too low under nonstress conditions.

Two types of corticosteroid receptors are important in the negative feedback mechanism: the type I or MR and the type II or GR. MR and GR are both found in varying densities within hypothalamic and hippocampal regions, with highest levels of GR in the hypothalamic PVN and the CA1 region of the hippocampus, whereas MR is found at high levels in all hippocampal CA regions (greatest in CA2) with lower but significant amount in the PVN.^35^ MRs have a high binding affinity (Kd) for GCs reportedly in the range of 0.1 nM and receptors are predominantly occupied by the lower levels of GCs found during basal secretion.

Meanwhile GR possesses a binding affinity that is 10-fold less (Kd = 1–2 nM) than MR and becomes mainly bound during significant elevations of GCs (e.g., following acute stressor).^36^ This is an important mechanism to extend the range of sensitivity to circulating CORT levels, which can undergo wide excursions in amplitude depending on the time of day and the environment in which the animals are facing. Many studies report sex differences in MR and GR as potentially contributing to more blunted negative feedback in females. Reduced density of GR and MR in the hypothalamus and hippocampus corresponds with the weaker negative feedback on the HPA axis in females and higher basal CORT secretions.^37^

Stress studies in rodents also show females with reduced GC binding and attenuated upregulation of GR in the hypothalamus^38,39^ following acute stressors, potentially resulting in a less robust negative feedback.

### Androgens and their steroid hormone receptors

The potent actions of T and DHT to suppress the HPA axis stress response^40–42^ work through binding to ARs (Fig. 1). Moreover, metabolites of T and DHT can additionally bind ERs, gamma-aminobutyric acid, and other receptors to induce changes in the HPA axis^43–46^ (Fig. 1). Hence, it is important to consider studies involving AR-deficient rodents to confirm the role of AR in altering stress reactivity.^47^ In rodent studies examining testicular feminization mutation (Tfm), AR is rendered mostly nonfunctional. Tfm rat males exhibit higher levels of T than wild types, but still have elevated CORT following acute stress.

Experiments in Cre-lox AR knockout mice with induced testicular feminization mutation (iTfm) further demonstrate increased basal and stress-induced CORT in iTfm mice treated with T than wild-type mice treated with T.^47–49^ iTfm mice also display increased anxiety-like behavior in light/dark box, open field, and elevated plus maze assays,^47–50^ implicating a role for AR in mediation of stress-related behavior. Williams et al.^51^ recently demonstrated a T-dependent reduction in anhedonia-like behavior with subchronic variable stress. Anhedonia-like behavior is also increased in AR-deficient rodents following chronic stress.^52^

Taken together, these data further emphasize the important role played by AR for androgenic suppression of the HPA axis. In humans, androgens are similarly thought to enhance mood through actions at the AR. Studies in prostate cancer patients demonstrate that treatment with flutamide increases depression symptoms.^53–55^ Moreover, another study by Wang et al.^56^ showed that males with complete dysfunctional AR with androgen insensitivity syndrome exhibit increased rates of depression.^57^

Androgens regulate HPA axis function through actions on estrogen receptor alpha (ERα) via metabolites of T, such as its conversion of T to E2 by aromatase (Fig. 1). This contrasts with AR-mediated inhibition of the HPA axis. Treatment with the selective ERα agonists, propylpyrazoletriol (PPT) and moxestral, increased levels of ACTH and CORT after stress in both sexes of rats.^58^ Studies also report the effect of ERα on the inhibition of negative feedback to the HPA axis, a more female-typical phenotype. Central implants of PPT near the PVN increased the diurnal peak of CORT and stress responsive increases in CORT and ACTH, while the estrogen receptor beta (ERβ) agonist, diarylpropionitrile (DPN), decreased stress hormone levels.^59^

Binding of ERα is generally thought to drive anxiogenic behavior in rodents. Pharmacological stimulation with PPT increases anxiety-like behavior.^60,61^ Downregulation of ERα induced by delivery of an adeno-associated viral vector into the medial preoptic area and posterodorsal amygdala of GDX rats showed decreased anxiety-like behavior in open field and light/dark box tests.^62^ Global knockout of ERα does not appear to influence anxiety-like behavior in female mice,^63^ but increases it in males,^64^ indicating possible sex differences in the actions of ERα. Such data suggest a potential role for ERα in stimulating the neuroendocrine stress response and related anxiogenic mood disorders mediated by the HPA axis activity.

Androgens also influence the HPA axis actions by E2 on ERβ. Metabolites of DHT such as 5α-androstane-3β, 17β diol (3β-diol), have relatively high binding affinity for ERβ,^65,66^ and numerous reports suggest an inhibitory role for ERβ on the HPA axis and stress-related behaviors (Fig. 1). Central administration of DPN, a selective ERβ agonist, diminishes ACTH and CORT stress responses in male and female rodents,^58,59,67,68^ but has no effect in ERβ knockout mice.^69^ GDX adult males further show a suppression of stress-induced ACTH and CORT by 3β-diol.^58^ Tamoxifen, a nonselective ER antagonist, when coadministered with DHT, minimized the suppressing effects of DHT on stress-induced CORT and ACTH.^58^

These data suggest a blockade of 3β-diol action at ERβ, which drives the suppression of the neuroendocrine stress response. Unlike ERα, ERβ has anxiolytic behavior effects. In male and female rodents, central implants of ERβ agonists, DPN and WAY-200070, decrease anxiety-like behavior in the open field and elevated plus maze tests.^56,68^ Hence, androgens may suppress anxiety-like behavior mediated through actions of 3β-diol, and binding to ERβ. Reports further demonstrate that 3β-diol does not alter anxiety-like behavior in ERβ knockout mice.^70^

Such data indicate that the effect of 3β-diol on anxiolytic behavior depends on functional ERβ. In support, ERβ knockout mice show increased anxiety-like behavior in open field and elevated plus maze in females,^63^ and increased depressive-like behavior in sucrose preference following inescapable foot shock in males.^71^ Data suggest that the lack of ERβ increases susceptibility for stress-related anxiogenic behaviors. In humans, the role of ERβ has been examined to a much lesser extent. Individuals with variations to the *Esr2* allele, rs1256049 and rs4986938, reported to experience increased major depression disorder and anxiety disorder, predominantly in females.^72,73^

## Androgens and Neuropeptide Systems in the Stress Response

### Dopamine

DA is a catecholaminergic neurotransmitter synthesized in the medulla of the adrenal gland that is responsible for modulating the HPA-axis alongside 5-HT and norepinephrine. DA also has a key role in the pathogenesis of schizophrenia and Huntington's disease, where high DA levels or DA receptor sensitivity contributes to schizophrenia and Huntington's development, and low brain levels of DA have been associated with causing Parkinson's disease.^74,75^

In addition, DA plays a role in reward and motivation responses, where a decrease in DA correlates to depressive-like symptoms including lack of motivation and loss of interest.^76^ In response to stress and CRH production, DA levels and dopaminergic neuronal activity increase in the mesolimbic DA system (MDS).^77^ Acute, short-term stressors in rodents (e.g., tail pinch, predator odor, immobilization) resulted in immediate significant increases in DA levels in the mesolimbic pathway.^78,79^

Comparatively, chronic stressors (e.g., food and water restriction, damp home cage bedding) in rats were associated with decreased DA levels or dampened DA neuronal activity.^80,81^ This difference suggests that when acutely stressed, high DA levels strengthen the motivation to escape, but when chronically stressed, low DA levels are associated with a maladaptive stress-induced depression. DA receptors, specifically DA receptor 1 (D1) and DA receptor 2 (D2), also play a role in maintaining activation of the HPA-axis poststress, as rats who were given specific D1 and D2 antagonists showed lower and shorter lasting periods of HPA response to a postimmobilization stressor.^82^

Gonadal hormones work to modulate DA levels where E2 is a negative influencer and T is a positive influencer of DA.^83,84^ Reduction of circulating E2 following ovariectomy in adult female rats resulted in greater DA transporter binding levels and D2 density in the MDS compared with their intact control rats.^83^ E2 has a biphasic mechanism involving a downregulation of D2 binding in response to an acute administration, and an upregulation of binding after chronic treatment.^84^

In contrast, T contributes to stimulating DA synthesis and metabolism, where midbrain DA neurons in male rats express ERs and ARs and are responsive to gonadal steroids.^85^ In a GDX study, DA-dependent spatial and learning tasks (e.g., lever pressing for a water reward) had lower breakpoints compared with control intact animals, where supplementation of T propionate attenuated the effects.^86^ In addition, GDX animals showed a depletion of medial prefrontal DA innervation in relation to their intact control group,^86^ implicating the role of T in DA pathways.

It has been suggested that treatment of T propionate acts as a protectant to dopaminergic neurons to age-induced oxidative damage in male rats.^87^ Such data indicate that androgens support the production of DA, while decreased T and DA levels correlate with increased risk for stress-related neuropsychiatric disease.

### Corticotropin-releasing hormone

Corticotropin-releasing hormone signaling via corticotropin-releasing factor receptor 1 (CRFR1) and corticotropin-releasing factor receptor 2 (CRFR2) in the pituitary is generally thought to regulate ACTH secretion.^88^ CRH secretion and binding to CRFR1 and CRFR2 have been demonstrated to mediate HPA axis responses and stress-related behaviors.^89^ In support of this, CRFR1 knockouts or CRFR1 antagonists suppress the HPA axis stress response and reduce anxiety- and depressive-like behaviors,^90^ while CRFR1 stimulation does the opposite.^91^ Unlike CRFR1 deletion, deletion of CRFR2 increases anxiogenic behavior and the HPA axis stress response.^92,93^

Sex differences have been observed in the roles of CRFR1 and CRFR2 in varying regions of the brain. There are higher levels of CRFR1 in the male PVN compared with females, with a decrease in CRFR1 in male PVN to female levels after GDX.^94–96^ Androgens have also been shown to upregulate CRFR2 in various brain regions. DHT propionate (DHTP) administration increases CRFR2 expression levels within the hypothalamus, hippocampus, and lateral septum (LS).^97^ Taken together, these data demonstrate that androgens decrease the HPA axis response and stress-related behaviors.^98,99^

The presence of AREs or estrogen response elements (EREs) in the promoter region of the *Crh* gene and its receptors allows androgens to directly alter the expression of CRFR1 and CRFR2.^100,101^ CRH and CRFR1 expressing neurons have also been shown to coexpress ARs and ERs.^94^ For instance, in rats and mice, CRH neurons in the PVN coexpress ERβ,^102^ suggesting that androgens may induce effects on the HPA axis through EREs in the upstream regulatory regions of CRH. Reports further show high coexpression CRFR1 and AR in PVN cells.^96^ While few neurons express both CRH and AR in the PVN, there is a large percentage of coexpression in the bed nucleus stria terminalis (BnST).^94^

Various studies in rodents additionally demonstrate that androgens can mediate CRH expression in the brain. For example, one study in males showed an increase in CRH 3 weeks after GDX. Androgen supplementation with DHT reversed this effect.^103^ DHTP treatment in GDX males further reduced PVN CRH expression following restraint.^60^ In contrast, CRH levels in the dorsolateral BnST have been shown to decrease following GDX of male rats and these effects were reversed with androgen treatment.^104^

Seale et al.^105^ reported a reduction in CRH cell expression in the female adult BnST when they were provided with neonatal T supplementation, suggesting that adult CRH levels are also influenced by neonatal androgens. These findings supported the concept that androgens suppress CRH expression in the PVN and ultimately the HPA axis response to stressors, potentially a mechanism that leads to lower depressive- and anxiety-like behaviors.

### Serotonin

5-HT is a monoamine neurotransmitter that stems from the median and dorsal raphe nuclei of the brainstem to stimulate the HPA-axis and stress response via directly activating CRH neurons in the PVN, increasing ACTH production and CORT release.^106,107^ In particular, the 5-HT receptors 5-HT1A and 5-HT2A have high degrees of colocalization in the PVN CRH neurons where agonists of these receptors resulted in increased ACTH secretion.^108^ Review articles have summarized the complex relationship between the 5-HT receptor subtypes, where agonist actions at specific 5-HT receptors (i.e., 1A, 1B, 2C, 4, 6) and blocking others (i.e., 2A, 2C, 3, 6, 7) produce antidepressive behaviors comparable with selective 5-HT reuptake inhibitors.^109,110^

Low levels of 5-HT have been associated with numerous illnesses, including anxiety and depression. G-protein-coupled ERs desensitize the 5-HT receptor signaling.^111^ Specifically, E2 actions at ERβ lead to modulation of the expression of 5-HT neurotransmitters via enhancing the expression of tryptophan hydroxyase-2, the rate-limiting enzyme in 5-HT synthesis, and decrease HPA-axis activation with lowered despair-like responses to stressors.^112,113^

With respect to sex differences, female mice had greater CORT production after administration of a selective 5-HT reuptake inhibitor—an effect that could be attenuated by T.^114^ E2 has shown a positive relationship between cortical 5-HT receptor binding in men, whereas T had no direct effect.^115^ Moreover, hormone replacement therapy administered to postmenopausal women improved 5-HT receptor binding and 5-HT signaling.^116^ Regarding T, there are different proposed mechanisms regarding the ability of this hormone to modulate 5-HT.

In one study, T was shown to be negatively associated with global 5-HT_4_R levels, which led authors to suggest that higher T levels correlated with a higher cerebral 5-HT level at baseline.^117^ However, 5-HT_4_R expression has been associated with low 5-HT and antidepressant-like behavior, which is in opposition to the conclusions of Perfalk et al.^117^

A study by Kranz et al.^118^ also concluded that a treatment of high-dose T in transgender men resulted in an increased 5-HT reuptake transporter binding and expression. The authors also proposed that T acts indirectly on serotonergic neurons by first converting to E2, as ERβ has been localized in serotonergic neurons, while ARs have not.^118^ Therefore, while E2 produces higher levels of 5-HT and CORT, and stimulation of the HPA-axis, the specific actions of AR stimulation by T or DHT have not been fully elucidated.

### Arginine vasopressin

The nonapeptide, AVP, is produced in hypothalamic neurons found in the PVN, BnST, supraoptic nucleus, and medial amygdala (MeA). When released to the general circulation via the posterior pituitary gland, the primary functions of AVP are to increase water reabsorption in the kidneys, and to constrict arterioles resulting in a higher arterial blood pressure. The release of AVP also affects behaviors related to anxiety and depression as it has been associated to work in conjunction with CRH to modulate the production of ACTH.^119,120^

In adult rodents, the number of AVP-expressing cells in the BnST and MeA is greater in males than females, while the number of AVP-expressing cells in the PVN was comparable between sexes.^121,122^ Studies also show that gonadal steroid hormones can modulate PVN AVP expression resulting in a greater number of AVP neurons in the certain brain areas of males compared with females.^104,123^ For example, implanting DHT into the BnST increased AVP PVN levels, whereas the introduction of hydroxyflutamide, a nonsteroidal antiandrogen, caused a decrease in PVN AVP expression.^123^

It has been hypothesized that in rats, since there is a low population of AR in PVN AVP neurons, androgens likely act indirectly to regulate expression via other brain regions such as the BnST and MeA.^124^ In both these areas, AVP neurons coexpress ARs and ERs, and therefore, these can provide direct regulation by androgens.^125^ Alternatively, because the PVN AVP neurons express ERβ, it could be argued that androgen metabolites such as 3β-diol can also act on the neurons directly.^126,127^ Studies using *in vitro* reporter gene assays show that 3β-diol can directly upregulate AVP promoter activity through binding to ERβ.^128^

Androgens have also been reported to modulate depressive-like behaviors and stress responses via directly promoting AVP neurons in the LS.^129^ Singewald et al.^130^ demonstrated that the LS is also a key region contributing to androgen inhibition of the HPA axis. For example, when rats had increased activation of AVP neurons in the LS, they were shown to have reduced immobility compared with control animals in the forced swim test,^129^ suggesting that AVP neurons in the LS can play a role in regulating and improving depressive-like behaviors and the response to androgens.

### Oxytocin

OT, a nonapeptide closely related to AVP, is produced in the hypothalamic PVN and SON and influences reproductive, postpartum, and social behavior, as well as playing a role to suppress the HPA axis and associated stress responses.^131–133^ Central infusion of supplemental OT diminishes PVN activation and secretion of ACTH and CORT, leading to an overall decrease in anxiety-like behaviors after stressors.^134,135^ Antagonizing OT receptors does the opposite and activates the PVN and increases anxiety-like behaviors.^132^ Moreover, OT receptor knockout male mice had an overactivation of the HPA axis following stress,^136^ supporting the role of OT in reducing the HPA axis activity.

ARs have been colocalized with OT in the medial parvocellular region of the hypothalamic PVN.^124^ OT has also been shown to be directly regulated by the androgen metabolite, 3β-diol, using *in vitro* reporter gene assays where 3β-diol and ERβ were transfected into human and rodent cell lines.^137^ These effects were traced to a composite response element lying in the proximal OT promoter.

*In vivo* studies show that GDX male rats administered T propionate had significantly higher amounts of OT release from PVN neurons and subsequent binding in the ventromedial hypothalamus (VMH) and BnST.^138^ It could be hypothesized that estrogenic metabolites of androgens regulate OT neuron function through an action mediated by ERβ, whereas receptor numbers are regulated through ERα.

Patisaul et al.^139^ demonstrated that treatment with E2 and progesterone in GDX female mice increased OT transcripts in the PVN, suggesting an estrogen-dependent role of ERβ in OT regulation. Treatment with E2 also enhanced OT receptor binding in the MeA and VMH in mice and rats.^139^ Interestingly, in the MeA and VMH, regions where ERα is predominantly expressed at higher levels than ERβ, OT receptor binding in ERβ knockout and wild-type mice was similarly increased following E2 treatment.^139^ This indicates that ERα is essential for the regulation of OT receptors in these regions, while ERβ does not appear to play a role. Moreover, OT antagonists minimize effects of the ERβ antagonist DPN on anxiety-like behaviors.

Taken together, these data indicate that androgen-mediated OT production and binding potentially suppress the HPA axis through these mechanisms, which can influence anxiolytic behaviors and attenuate stress-related responses.^140^

## Androgens and Their Role in Therapeutic Treatments

The physiological response to acute stress is beneficial and enhances cognition, immune function, and metabolism to increase chances of survival.^18^ For instance, short-term release of GCs (cortisol in humans, and CORT in rats and mice) can induce gluconeogenesis to break down glucose stores and provide the proper nutrients and energy in response to an acute stressor.^141^ In contrast, chronic activation of the stress response has deleterious effects on immune system activity and neurotoxicity. Such long-term insults to the HPA axis stress response ultimately increases risk for cardiovascular, immune, metabolic, and neuropsychiatric diseases.^142^

These stress-related disorders arise differently in males versus females, given that the sex differences have been reported in the function of the HPA axis.^143,144^ For example, males are two to three times less likely to develop depression than females^145^ and exhibit decreased subclinical symptoms and decreased rates of comorbid anxiety due to increased circulating levels of T.^146,147^ Similarly, men with prostate cancer undergoing androgen-deprivation therapy present with increased stress-related disorders, including anxiety and depression.^148^ Male rats display reduced anxiety- and depressive-like behaviors compared with females due to elevated levels of androgens, namely T.

A large body of research implicates androgens in the attenuation of the integrated central stress response. Therefore, a role for androgens in the treatment of stress-related neuropsychiatric disorders (Fig. 1), such as depression and anxiety, is emerging.^29,149^

Testosterone replacement therapy (TRT) is a common treatment for hypogonadal men. A recent meta- analysis found that men diagnosed with hypogonadism undergoing TRT presented with decreased depressive-like symptoms and improved mood.^150,151^ Moreover, men with diabetes mellitus type 2 and hypogonadism in a double-blinded placebo study received intramuscular TRT or a placebo for 30 weeks.

Subjects were evaluated by the Aging Male Symptom Scale, based on the Hospital Anxiety and Depression Scale and Global Efficacy Questionnaire to evaluate overall mood. Scores were significantly increased in those who received TRT in contrast to the placebo group, implicating the role of T in attenuating depressive- and anxiety-like symptoms.^152^ Such effects of T treatment are further demonstrated in animal models. In rodents, T administration increased the synthesis and release of 5-HT from the dorsal raphe nuclei and increased neuroplasticity in the hippocampus to induce antidepressive-like states and improved mood.^153^ T treatment also reduced anxiety and depressive-like behavior in male rodents.^154–156^

Hence, the correlation between T and anxiolytic activity suggests high efficacy of T therapeutics in treating stress-related disorders. Although TRT has been shown to be efficacious in hypogonadal men in improving mood disorders, evaluation of long-term health risks will be important.

Administration of DHEA and its metabolite, DHEA sulfate, has also been proposed for the treatment of neuropsychiatric disease. Levels of DHEA and DHEA-sulfate decrease with age, leading to fatigue, anxiety, and depression.^157^ Evidence further suggests that there is a negative correlation between plasma DHEA and DHEA sulfate levels and cortisol in stress and anxiety.^158,159^ Supplementation with these compounds increases androgen levels, attenuates stress-induced cortisol output, and improves mood disorders.^160^

In human studies, DHEA or DHEA sulfate displayed improvement in anxiety and depressive symptoms.^161,162^ To support these findings, DHEA and DHEA-sulfate doses decreased depressive-like symptoms and enhanced cognition in patients after 6 months of administration. Interestingly, depressive-like symptoms and cognition worsened following the withdrawal of treatment.^157^ A meta-analysis of randomized-controlled trials further demonstrated that DHEA treatment had a beneficial effect on depressive symptoms in 853 females and male subjects. Side effects from DHEA treatment were uncommon and transient in trials.^162,163^ Such data suggest an important role of DHEA in affective mood disorders and a promising outlook on DHEA as a therapeutic.^162–164^

The AR is an additional target for the therapeutic use of androgens. Selective AR modulators (SARMs) were first discovered near the end of the 20th century.^165^ SARMs are small-molecule drugs engineered to selectively bind AR in target tissues. The tissue type allows the ligand to exert both antagonistic and agonistic effects based on the types of coregulator proteins and cofactors present.^166^ In contrast, TRT is often associated with numerous off-target effects due to the lack of tissue selectivity that occurs with classical steroid treatment.^167,168^ The tissue-specific effects of SARMs make them an ideal candidate for androgen-based therapeutics.^167^

Androgen modulators are being studied as a potential treatment of cognition and mood disorders, including anxiety and depression. GDX male mice treated with a SARM for 4 months displayed enhanced cognition in the Morris water maze test. Chronic SARM treatment further decreased anxiety-like behavior in the elevated plus maze and open field tests.^169^ Such data implicate SARMs as a potential therapeutic for stress-related disorders. However, the development of SARMs is still in the early stages and undergoing clinical trials.^170–172^ Further studies are necessary to examine their efficacy and safety, but they remain a promising strategy for androgen therapy.^173,174^

## Conclusions

Androgens are an important factor to consider when examining sex differences in the HPA axis and stress-related behaviors. Several studies have been performed to assess the mechanism of action of androgens and androgen metabolites and their receptors involved in HPA axis regulation. These support their roles in driving sex-specific HPA axis phenotypes. It is important to consider that androgens and androgen metabolites exert varying hormonal and behavior effects depending on the brain region in which their associated receptor is located.

Future studies that examine the sites of these actions would be beneficial to understanding the role androgens play in the stress response. Abundant evidence additionally supports a role for androgens in neuropeptide systems that interact with the HPA axis (CRH, AVP, OT, 5-HT, and DA), but precise circuitries remain undescribed. Further research in these areas will fill these gaps in our knowledge in how steroidal gonadal hormones contribute to sex differences in important stress regulatory systems and related neuropsychiatric disorders. Moreover, therapeutic methods involving androgens and SARMs present a positive outlook.

Androgen replacement therapies such as TRT are clinically demonstrated to successfully attenuate stress disorders, such as anxiety and depression, in males and females. However, due to the potential adverse off-target effects of TRT, SARMs have become more of an interest in the present field of androgen therapies. SARMs are chemically engineered to target specific tissues expressing ARs, allowing them to be better tolerated and highly selective.

However, while SARMs have been studied in several Phase I and Phase II clinical trials,^170–172^ and pre-clinical data suggest a positive outcome, they are not yet FDA approved. Nevertheless, SARMs appear to have great potential for the revolutionary treatment of numerous androgen-mediated medical challenges.

## Abbreviations Used

17β-HSD: 17β-hydroxysteroid dehydrogenase
3α-diol: 3α-androstanediol glucuronide
3β-diol: 5α-androstane-3β, 17β diol
3β-HSD: 3β-hydroxysteroid dehydrogenase
5αR: 5α-reductase
5-HT: serotonin
ACTH: adrenocorticotropin hormone
AR: androgen receptor
ARE: androgen response element
AVP: arginine vasopressin
BnST: bed nucleus stria terminalis
CBG: corticosteroid binding globulin
CORT: corticosterone
CRFR1: corticotropin-releasing factor receptor 1
CRFR2: corticotropin-releasing factor receptor 2
CRH: corticotropin-releasing factor
DA: dopamine
D1: DA receptor 1
D2: DA receptor 2
DHEA: dehydroepiandrosterone
DHT: dihydrotestosterone
DHTP: DHT propionate
DPN: diarylpropionitrile
E2: estradiol
ER: estrogen receptor
ERE: estrogen response element
ERα: estrogen receptor alpha
ERβ: estrogen receptor beta
GABA: gamma-aminobutyric acid
GC: glucocorticoids
GDX: gonadectomy
GR: GC receptor
GREs: GC response elements
HPA: hypothalamic/pituitary/adrenal
iTfm: induced testicular feminization mutation
LS: lateral septum
MDS: mesolimbic DA system
MeA: medial amygdala
MR: mineralocorticoid receptor
OT: oxytocin
PPT: propylpyrazoletriol
PVN: paraventricular nucleus of the hypothalamus
SARM: selective AR modulator
SHIP: short heterodimer partner
T: testosterone
Tfm: testicular feminization mutation
TRT: testosterone replacement therapy
VMH: ventromedial hypothalamus
